# A quality improvement program to reduce the time on the lung transplant waiting list at the Nantes University Hospital

**DOI:** 10.1186/s13023-017-0748-4

**Published:** 2018-02-08

**Authors:** Isabelle Danner-Boucher, Véronique Loppinet, Aurore Boxus, Claire Dary, Anne Brigitte Lambert, Marine Prieur, Céline Vallet, Adrien Tissot

**Affiliations:** Pulmonology Department, Thorax Institute, Nantes University Hospital (NHU), Nantes, France

**Keywords:** Cystic fibrosis, Quality improvement program, Lung transplantation, Lung transplant list, Waiting time on lung transplant list

## Abstract

**Background:**

In 2010, the time on the lung transplant waiting list in Nantes University Hospital (NUH) was 9.2 months, compared to a French national median of about 4 months. The NUH transplant unit performs both heart and lung transplantations, which can be seen as competing activities. To fix the problem, the adult Cystic Fibrosis (CF) team decided to engage in the French CF Quality Improvement Program (QIP PHARE-M) in 2012. The objectives were: i) To reduce the time on the lung transplant waiting list at the Nantes Transplant Unit by increasing the number of lung transplants per year twhile maintaining a 5-year survival rate above the French national average. ii) To improve the organization of the lung transplant access process and the quality of the waiting time for patients.

**Methods:**

A quality controller was involved as the QIP referent to coach the CF quality team, analyze the pre-transplant process, and set up meaningful measures. Benchmarking was performed with other transplant units, and staff discussions were held with the Transplant Team (TT) to assess the outcomes of rejected donor lungs. Negotiations were made with the hospital administration. Plan, Do, Study and Act cycles were used to redesign the pre-transplant assessment in connection with the CF centers (CFC) referring patients to the NUH transplant unit.

**Results:**

i) The flow of patients has been reorganized, decreasing the time spent in surgical intensive care by increasing the number of beds in the intensive care unit, and a chest physician has been recruited ii) The number of organs rejected has been reduced iii) Lung transplant activity has increased to 20–25 transplants per year, and the median waiting time was reduced to 3.5 months for patients transplanted in 2014 and to 1.85 months for patients transplanted in 2015 iv) Added-value activities including education, information, and psychosocial support are now offered to patients during the waiting time.

**Conclusion:**

The QIP PHARE-M, including coaching by a quality-engineer, has helped our adult CF center address its specific lung transplant issues and redesign the lung transplant process for both local patients and patients referred by other CFC.

## Background

The French national median time on the lung transplant waiting list reported by the French Biomedical Agency (ABM) in 2012 was 4.4 months, while it was 9.2 months for the Nantes University Hospital (NUH). In 2010–2011, the lung transplant activity was at 15–20 transplants per year, including 60% in CF patients,

As the only transplant center in western France, our centre provides a much needed service for transplant particularly for remote areas where traveling to Paris for a transplant is logistically complicated given the time required to arrive at the transplant center during a call. Half of CF patients who have received a lung transplant in Nantes have been referred from other CF centers in France.

The surgery department is unique in that our surgeons at once practice lung transplants, heart transplants, and assisted circulation, as well as scheduled lung and heart surgery. These activities compete with each other, and certain necessary choices are made, not always in favor of lung transplants. On an ethical level, we felt that it was impossible to continue to work with such a discrepancy in our waiting times, including a risk of death on the waiting list greater than the French national average. The survival of our transplant center was at stake. When we joined the QIP PHARE-M, we decided to choose an objective that was original but close to our hearts: to reduce the time on the lung transplant waiting list in Nantes by increasing the number of transplants while maintaining the quality of patient management.

The primary objective was to reduce the time on the lung transplant waiting list at the Nantes Transplant Unit by increasing the number of lung transplants per year to achieve the objective of 30 transplants/year, while maintaining the quality of patient management and a 5-year survival rate above the French national average (55.7%). The secondary objective was to improve the organization of the lung transplant access process and the quality of the waiting time for patients, both those at our CFC and those referred by other CFCs, to better prepare them and better meet their needs.

## Methods

A working group was formed within the CF multidisciplinary team comprising one CF coordinator nurse, one physiotherapist, two psychologists, two pulmonologists, one patient referee who had not undergone a transplant, and one quality controller in charge of coaching the group and helping it analyze pre-transplant processes. This group, called the quality team, worked in accordance with the recommendations and techniques for quality improvement of the QIP PHARE-M. The quality team participated in four face-to-face sessions and six webinars. Secondly, two secretaries were included, their presence being required to manage pre-transplant reviews and the waiting list.

We prepared a fishbone diagram to list the different causes of the problem linked to the main headings: patients, professionals, material resources, other CFCs that refer their patients to us for a transplant, and management processes. An analysis of the transplant process was performed that described the different steps of the process, from pre-transplant to post-transplant follow-up: initial consultation, pre-transplant review, registration on the waiting list, and call for transplant. All these steps were the subject of a team reflection aimed at streamlining the process.

At the same time, information concerning waiting times was disseminated to raise awareness among the different players in the care chain (anesthetists/intensivists, surgeons, pulmonologists, and cardiologists) and negotiations were made with the hospital administration with the help of our Head of Department to alert the medical direction of the situation and ask for more support. The NUH transplant Unit had a reputation for being more demanding than most French centers regarding the quality of grafts accepted. A thesis written by Dr. T. Madjer examined the outcomes after 6 months of recipients of grafts that had been rejected at the NUH because they were deemed to be of poor quality, then accepted at another transplant center.

A satisfaction survey on the experience of the pre-transplant review (PTR) and then the transplant waiting time was sent to 40 patients who had undergone a transplant in the previous 3 years or who had done a pre-transplant review in the course of these previous 3 years. The aim was to gather these patients’ opinions on the points that could be optimized to improve the quality of their time on the lung transplant waiting list. The questionnaires were sent by email, with a secondary reminder by post. The results were analyzed anonymously.

Plan, Do, Study and Act cycles were described to structure actions for change, test them, and evaluate their results.

## Results

**Fig. 1:**
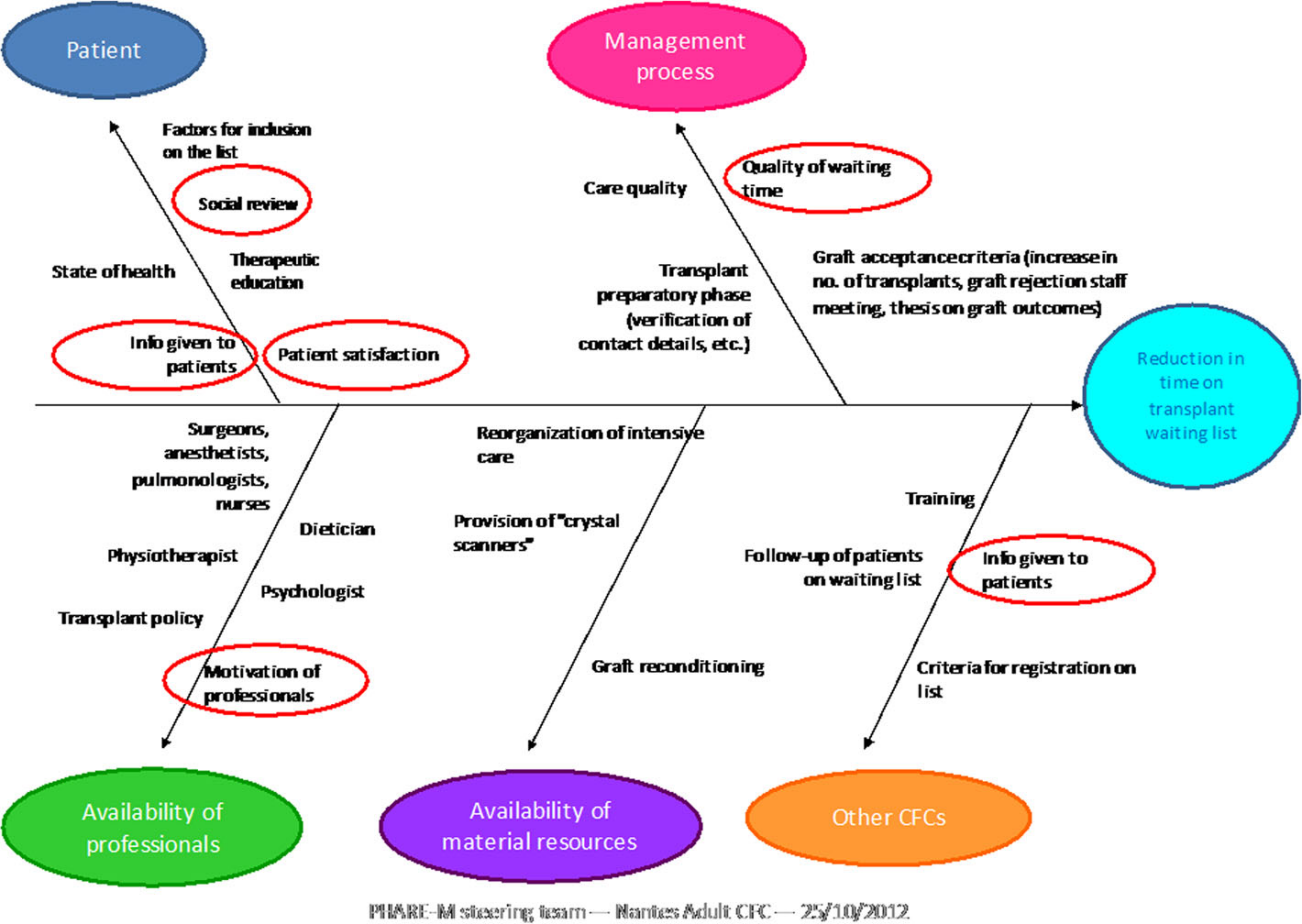
Fishbone diagram identifying the points that can be improved at the CFC and in the thoracic and cardiovascular surgery department at the Nantes CHU

The fishbone diagram identified the points to be improved at the CFC and in the thoracic and cardiovascular surgery department at the Nantes Hospital (Fig. 1).

All the players involved in the care journey developed a heightened awareness of the need to reduce the waiting period, accompanied by a renewed motivation to improve quality in the lung transplant process.

The thesis work showed that the FEV1 and survival of patients who had undergone a transplant at Nantes were comparable to those of patients who had undergone a transplant at another center with a graft that had been rejected in Nantes as a poor graft. These results allowed the team to expand its acceptance criteria slightly. At the same time, each donor lung rejected as a “poor graft” was discussed at the weekly transplant staff meeting, allowing contrasting opinions to be expressed. The surgeons agreed to adopt the volume reduction technique, which had not been practiced up to that time, to accept lungs that were morphologically too large and reduce their size (by lobectomy or peripheral resection) to render them morphologically suitable [1]. As a consequence, the rate of lung proposal refusal for volume missmatch decreased from 26% in 2010 to 21% in 2012.

**Fig. 2:**
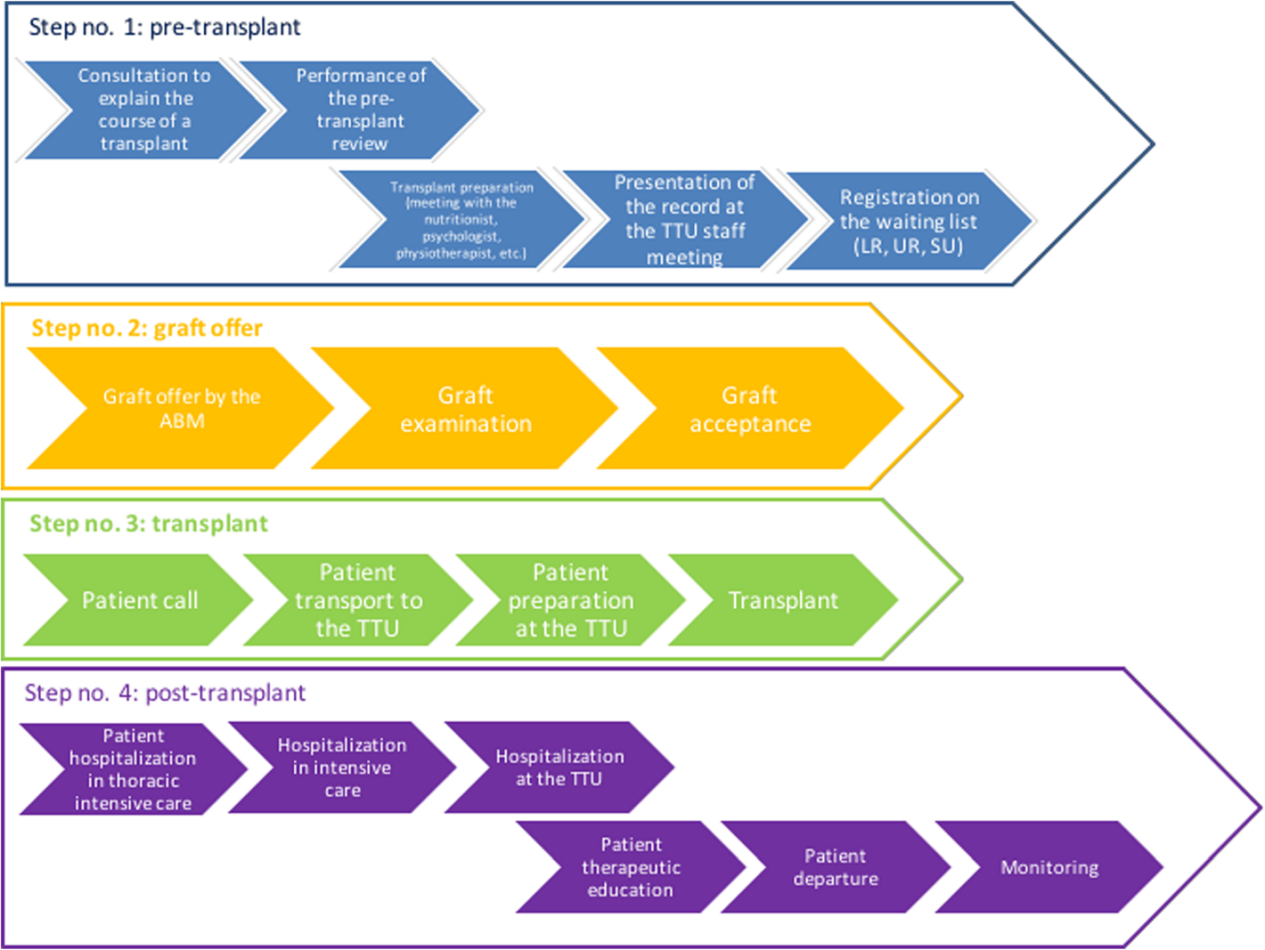
Transplant process from initial consultation to post-transplant follow-up. TTU: Thoracic transplant unit belonging to the thoracic and cardiovascular surgery department (10 beds), managed at once by anesthetists/intensivists, pulmonologists, and cardiologists

On the basis of the process described (Fig. 2), the hospital administration got involved to make the decision to allocate additional resources, namely:

- The opening of two additional intensive care beds;
- The reorganization of the downstream healthcare network to quickly move patients having undergone a transplant out of surgical intensive care and into pulmonology intensive care, to keep from compromising the schedule of surgeries in the operating room, which requires patients undergoing heart operations to stay 24 h in the surgical intensive care unit; and
- The acquisition of the machine required for ex vivo lung graft reconditioning, allowing the quality of certain lungs to be improved and allowing them to be transplanted when they do not meet the initial acceptance criteria. In theory, this will allow an increase in the number of usable grafts and thus the number of transplants performed. This technique is in the process of being acquired, and the staff are in the process of being trained [2, 3]. However, this technique was not in place at the time of the PHARE program.

**Fig. 3:**
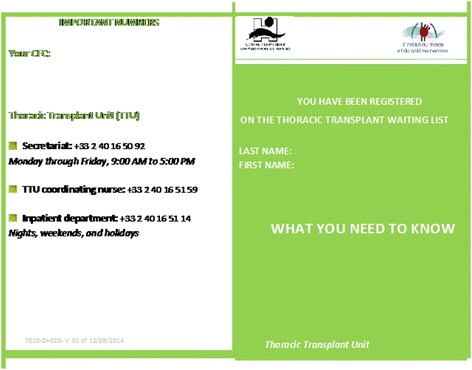
So-called “Memo Card” tool with essential reminders given to the patient at the time of registration on the waiting list

Certain patient education actions were carried out with the creation of tools such as the memo card (Fig. 3) so that a graft is not lost because of patient unavailability. The memo card reminded the patients of the instructions: to give notice in the event of a change in telephone number, to pay attention to their mobile phone battery, to notify the transplant team if they are hospitalized, and to stay up to date on their vaccinations and anti-HLA Ab monitoring (a lack of recent immunological monitoring necessitates a crossmatch, which can only be organized with a geographically close graft and can thus lead to a transplant being cancelled).

To keep from compromising care quality with an increase in the number of transplants and a corresponding increase in the follow-up load, post-transplant follow-up was reorganized with the other CFCs in the region. Alternating follow-up between our transplant center and the patient’s CFC of origin was thus established: it starts 1 year after the transplant and can be suspended at any time on the opinion of the transplant center if a problem is identified with the relay team. The transplant center remains the center responsible for the patient.

Several actions were undertaken to carry out this alternating post-transplant management.

- Theoretical training was conducted at all the relay centers, followed by immersion training of several days per team at our center.
- Alternating follow-up was progressively established with the CFCs in the region that refer their patients to us for a transplant, after the CFC teams were trained in the unique features of the follow-up of transplant recipients.
- Support was provided with the institution of a time for exchanges in the form of videoconferences one to two times per year with these teams.
- Our team traveled to meet with the main relay team, which strengthened relationships.

**Fig. 4:**
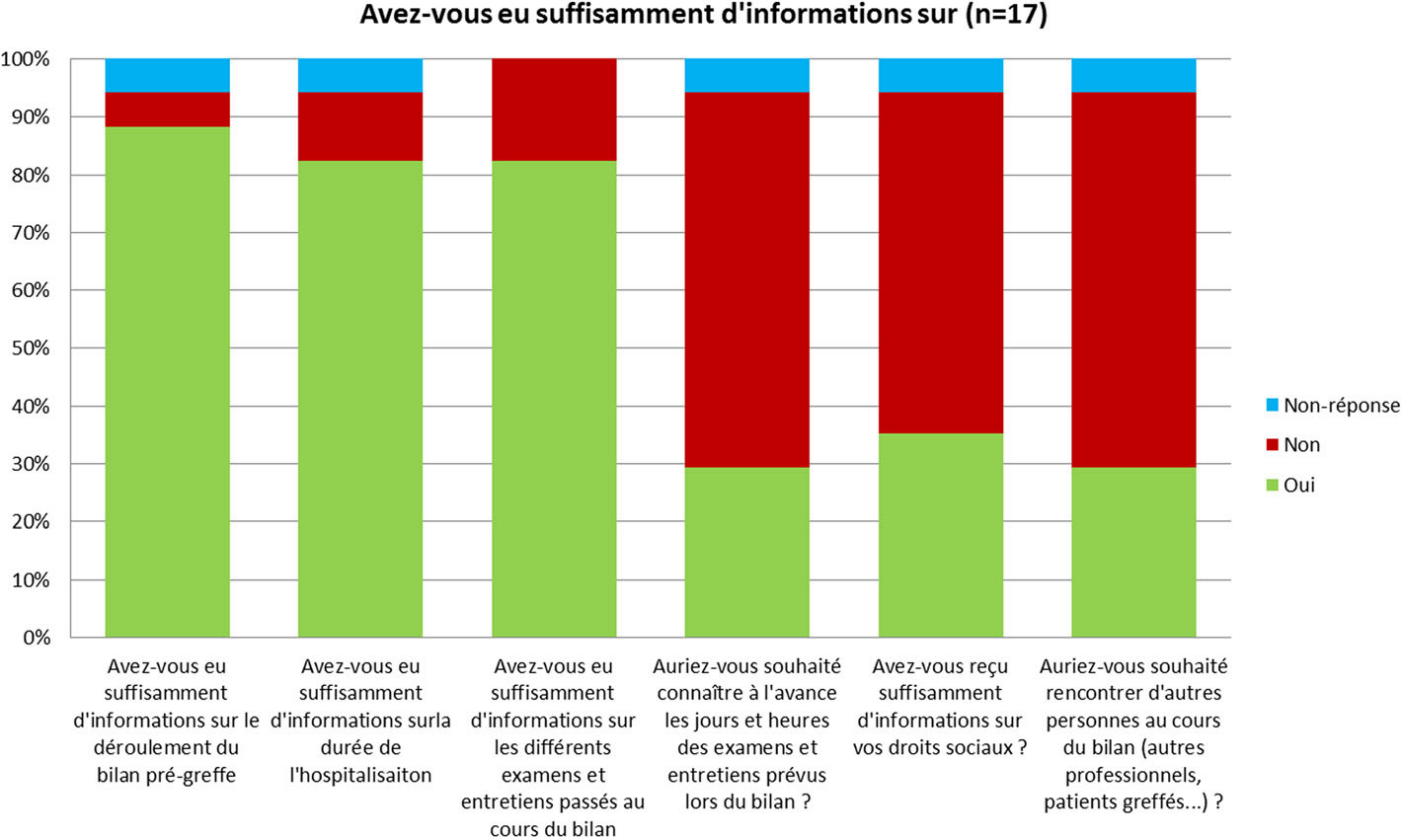
Graphic prepared based on the survey carried out in patients and showing their information needs based on the responses in the 17 questionnaires returned

The satisfaction survey on the experience of the pre-transplant steps and waiting time was sent to 40 patients who underwent a transplant, and 17 responses were collected. The survey showed good overall patient satisfaction. It essentially revealed a lack of information on social needs. Certain actions were established (Fig. 4):

- A tool to identify social needs was created, and
- A possible consultation with the social worker was scheduled.

This survey made us aware of the difference in transplant preparation between the pre-transplant patients followed up at our center and the patients followed up elsewhere and referred for discussion of a transplant. The latter all benefitted from an initial consultation with twice the usual time for exchanges to conduct an initial study of the record and give them information on the transplant process, its challenges, its risks, and the course of the care journey. By contrast, the patients followed up at our center received this information in the course of their consultations. However, the patients felt that this dedicated time to talk about the transplant, often with their relatives, was important. Thus, we established a clearly identified transplant information period for the pre-transplant patients followed up at our center in the form of an additional double-length consultation.

Following a review of our practices, each patient was assigned a referring physician, which had not been the case earlier, when the patients could be seen by different physicians in the course of the pre-transplant consultation, then the PTR week. This assignment of a head physician in charge of presenting each patient’s pre-transplant record at the transplant staff meeting and monitoring each patient’s subsequent evolution made the journey smoother.

We also instituted a PTR restitution consultation that had not been systematic before this study and that seemed necessary to us.

Since 2012, the time on our lung transplant waiting list has reduced considerably. The median waiting time for transplant recipients went from 9.2 months in 2008–2011 to 5.6 months in 2012–2013, then 3.5 months in 2014 and 1.85 months in 2015 (as of 31/07/2015).

## Discussion

When in 2011–2012 we became aware of the major discrepancy between our team’s median waiting time and the waiting time at other centers, we found it difficult not to talk about it with the patients on our list whose condition was the most severe. Some of them chose to leave our list to be registered at the Foch (Suresnes) center, which then had a median waiting time of around 1 month, well below the French national average. This departure of a few patients, combined with an increase in the number of transplants performed associated with the PHARE-M program, reduced the number of patients registered on our waiting list. We went from a list of around 20 patients to six in late 2015. Once the old patients who had been registered for a long time had disappeared from the list (following a transplant, death, or a transfer to another list), our waiting list was self-regulating, with comparable numbers of registrations and transplants per year.

**Fig. 5:**
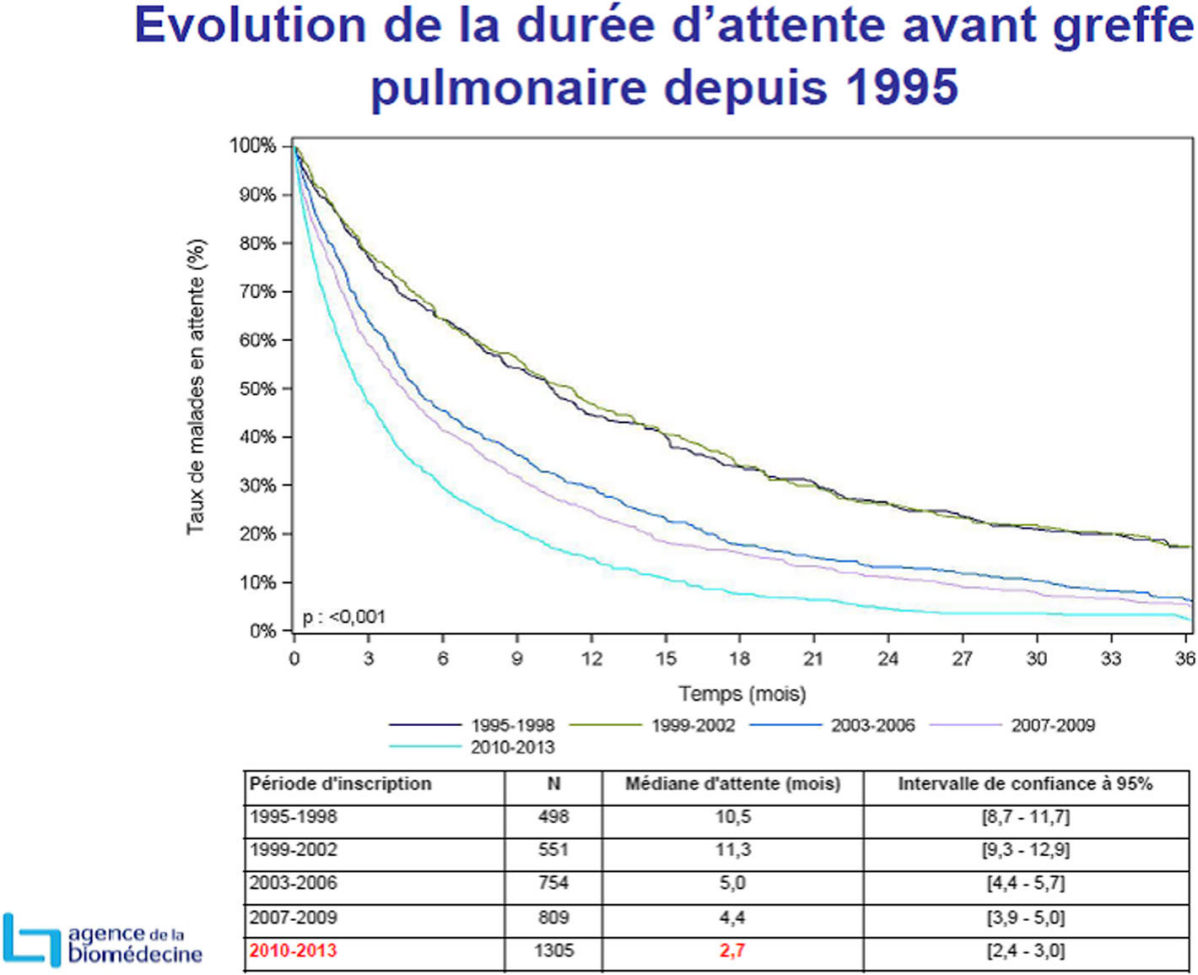
Graphic provided by the French Biomedical Agency on the changes in Time on lung transplant waiting list in 1995–2013

**Fig. 6:**
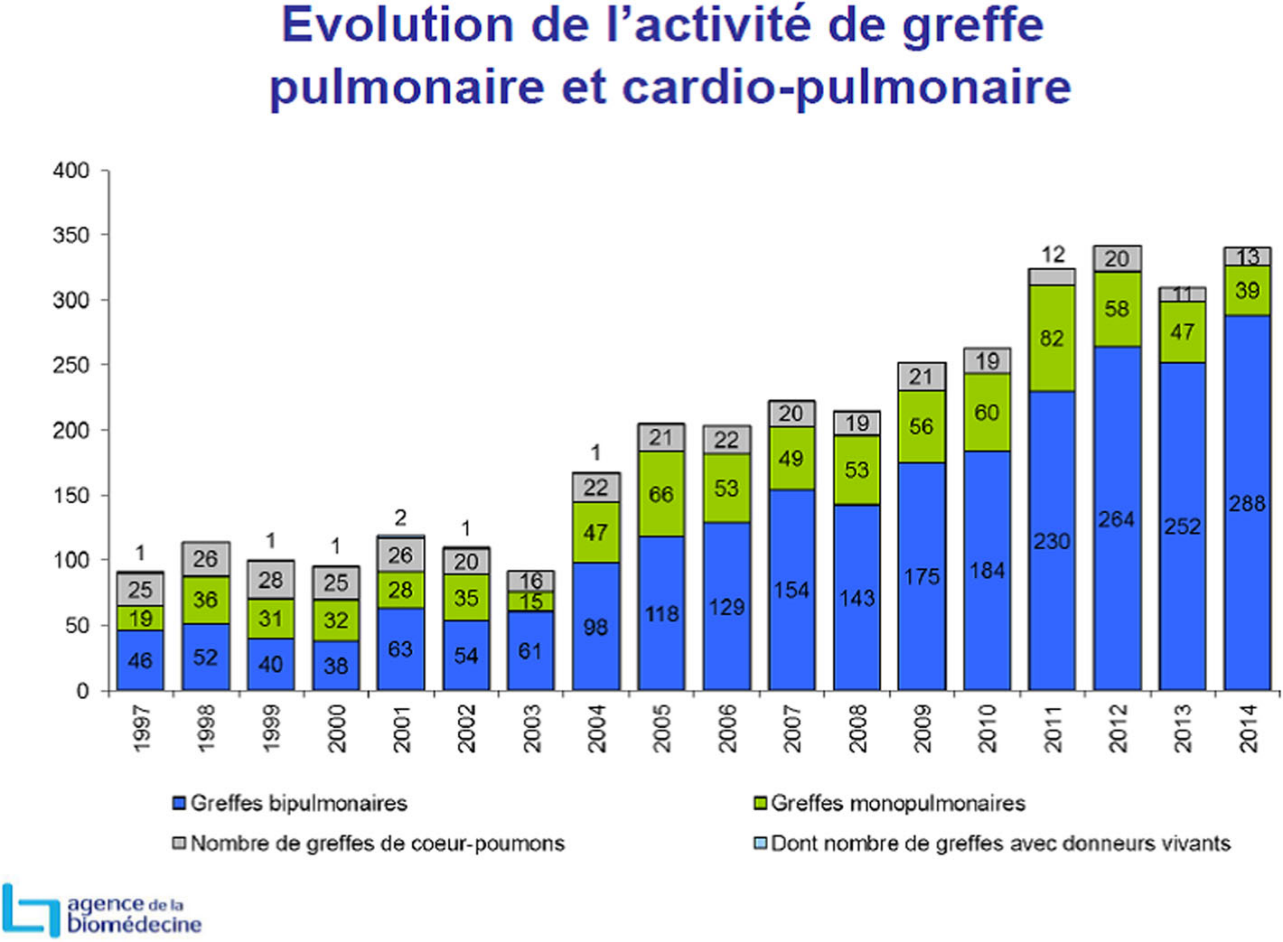
Graphic provided by the French Biomedical Agency on the changes in lung and heart–lung transplant activity in 1997–2014

This must be compared to the reduction in the French national median waiting time due to an increase in the number of transplants in France (Fig. 5). This was mainly linked to a work conducted on the expansion of the graft acceptance criteria that increased the French national number of transplants from around 180 double lung transplants in 2009–2010 to around 260 in 2012–1013 (Fig. 6).

It is important to note that the median figures reported by the French Biomedical Agency are always delayed, while the median waiting times reported for Nantes are real-time figures. Thus, the median waiting time given in 2012 by the Agency concerned the years 2007–2009. The median reported in summer 2015 concerned the years 2010–2013 and also reduced to 2.7 months (Table 1) [4].

**Table 1:** Changes in the acceptance and attribution of lung organs in Nantes Hospital in 2010–2015

	2010	2011	2012	2013	2014	2015
Lung proposal n	247	478	532	355	199	281
Acceptance n (%)	21 (9)	20 (4)	34 (6)	27 (8)	27 (14)	26 (9)
Refusal n (%)	224 (91)	458 (96)	498 (94)	327 (92)	172 (86)	255 (91)
Refus morpho n (%)	64 (26)	79 (17)	110 (21)	7 9 (22)	37 (19)	52 (19)
High emergency n	6	5	8	8	4	5
Listed patients n	18	10	19	16	21	19

It is important to balance these figures with several datas that could have impact our results. First, we saw a decrease of the refusal rate from 2011 (96%) to 2014 (86%) and the main reason for it is a significant decrease of refusal for morphological reason. Howerver, there was a remarkable variation over the past 6 years in the total number of lung proposed to our team: 247 in 2010, 478 in 2011, 532 in 2012 and 355 in 2014. This might be due to the implementation of extended donor criteria at that period and it clearly can have consequence on the analysis of our rate of refusal. Secondly, the number of patient who underwent lung transplantation under the High Emergency rule increased from five patients in 2010 to 8 patients to 2012. It dropped to 4 in 2014 and 5 in 2015. Finally, we saw a variation in the number of patients listed in Nantes (18 in 2010, 10 in 2011 and 19 in 2012).

The PHARE-M process includes patients in the working group. We asked a referent patient who had not undergone a transplant and whose state did not foreseeably require a transplant in the next 5 years to participate. The working sessions in which she participated were chosen deliberately on the basis of her interest and state of fatigue. This young woman observed that her participation had stirred up certain emotional reactions in line with the reality she faced in advance, despite the efforts made to choose a patient not expected to require a transplant for some time. However, she said that she appreciated this collaboration and found it enriching. Perhaps we should have chosen a patient who has already undergone a transplant, or included another patient, to further enrich the discussion around the experience.

## Conclusion

Our team was committed to participate in the PHARE-M improvement program recognizing the need to change in order to improve the service to our patients. With this in mind, our team reduced the median time on the lung transplant waiting list in Nantes. Now we are close to the French national average. The acquisition of the ex vivo lung graft reconditioning technique that is expected to start in early 2016 will position Nantes as a transplant center determined to continue this program with its associated technological innovations. This program also allowed us to review our management processes and qualitatively improve our patients’ waiting time and pre-transplant journey. Our program improved tremendously in all these areas and through this publication we would like to encourage other programs o work on similar or more difficult projects.
